# Can Exercise Training Alter Human Skeletal Muscle DNA Methylation?

**DOI:** 10.3390/metabo12030222

**Published:** 2022-03-02

**Authors:** Luis A. Garcia, Rocio Zapata-Bustos, Samantha E. Day, Baltazar Campos, Yassin Hamzaoui, Linda Wu, Alma D. Leon, Judith Krentzel, Richard L. Coletta, Eleanna De Filippis, Lori R. Roust, Lawrence J. Mandarino, Dawn K. Coletta

**Affiliations:** 1 Department of Medicine, Division of Endocrinology, University of Arizona, Tucson, AZ 85724, USA; lagarcia1@arizona.edu (L.A.G.); rocioz@deptofmed.arizona.edu (R.Z.-B.); bcampos9@email.arizona.edu (B.C.); almadleon@arizona.edu (A.D.L.); jkrentzel@arizona.edu (J.K.); mandarino@email.arizona.edu (L.J.M.); 2 Center for Disparities in Diabetes Obesity and Metabolism, University of Arizona, Tucson, AZ 85724, USA; richard.coletta@gmail.com; 3 Phoenix Epidemiology and Clinical Research Branch, National Institute of Diabetes and Digestive and Kidney Diseases, National Institutes of Health, Phoenix, AZ 85004, USA; samantha.day@nih.gov; 4 Department of Physiology, University of Arizona, Tucson, AZ 85724, USA; yhamzaoui@email.arizona.edu (Y.H.); lindawu@email.arizona.edu (L.W.); 5 Department of Endocrinology, Metabolism and Diabetes, Mayo Clinic Arizona, Scottsdale, AZ 85259, USA; defilippis.elena@mayo.edu (E.D.F.); roust.lori@mayo.edu (L.R.R.)

**Keywords:** exercise training, DNA methylation, euglycemic hyperinsulinemic clamp, skeletal muscle, insulin sensitivity, VO2 peak

## Abstract

Skeletal muscle is highly plastic and dynamically regulated by the body’s physical demands. This study aimed to determine the plasticity of skeletal muscle DNA methylation in response to 8 weeks of supervised exercise training in volunteers with a range of insulin sensitivities. We studied 13 sedentary participants and performed euglycemic hyperinsulinemic clamps with basal vastus lateralis muscle biopsies and peak aerobic activity (VO2 peak) tests before and after training. We extracted DNA from the muscle biopsies and performed global methylation using Illumina’s Methylation EPIC 850K BeadChip. Training significantly increased peak aerobic capacity and insulin-stimulated glucose disposal. Fasting serum insulin and insulin levels during the steady state of the clamp were significantly lower post-training. Insulin clearance rates during the clamp increased following the training. We identified 13 increased and 90 decreased differentially methylated cytosines (DMCs) in response to 8 weeks of training. Of the 13 increased DMCs, 2 were within the following genes, *FSTL3*, and *RP11-624M8.1*. Of the 90 decreased DMCs, 9 were within the genes *CNGA1*, *FCGR2A*, *KIF21A*, *MEIS1*, *NT5DC1*, *OR4D1*, *PRPF4B*, *SLC26A7*, and *ZNF280C*. Moreover, pathway analysis showed an enrichment in metabolic and actin-cytoskeleton pathways for the decreased DMCs, and for the increased DMCs, an enrichment in signal-dependent regulation of myogenesis, NOTCH2 activation and transmission, and SMAD2/3: SMAD4 transcriptional activity pathways. Our findings showed that 8 weeks of exercise training alters skeletal muscle DNA methylation of specific genes and pathways in people with varying degrees of insulin sensitivity.

## 1. Introduction

The physical demands of the body dynamically regulate skeletal muscle. It is a highly plastic tissue adaptable to many stimuli, such as contractile activity. Kirwan et al. recently discussed the metabolic effects of acute and chronic exercise changes [1]. Briefly, the acute effects of exercise (i.e., a single bout of exercise) include immediate improvements in blood glucose levels and also increased insulin sensitivity at the muscle tissue [1]. The chronic effects of exercise include increased skeletal muscle expression of genes coding for muscle fiber type, mitochondrial biogenesis, and glucose transporter 4 (GLUT4) proteins [1] in combination with insulin action improvements.

Exercise ultimately changes many molecular processes tightly regulated by transcriptional, translational, and post-translational mechanisms [2,3,4]. More recently, there has been an interest in the epigenetic changes in skeletal muscle following chronic exercise training. Waddington first coined the term epigenetics as heritable changes in gene function that occur without a change in nucleotide sequence [5]. The most commonly studied epigenetic mechanism is DNA methylation as a regulator of gene expression. For example, gene expression decreases when DNA methylation occurs at a gene promoter. DNA methylation is the addition of a methyl group to the carbon 5 position of the cytosines in DNA, and DNA methyltransferases tightly regulate this process.

Jacques et al. recently published a systematic review on the epigenetic changes that occur in healthy human muscle following exercise [2]. In that review, only a handful of studies measured DNA methylation changes in skeletal muscle following an exercise training program [6,7,8,9]. Even fewer studies have measured DNA methylation in skeletal muscle following exercise training in people with varying degrees of insulin sensitivity [6,10]. This area of research is clearly in its early stages and warrants additional investigation. Therefore, the focus of this study was to determine skeletal muscle global DNA methylation in a cohort of volunteers with a range of insulin sensitivities following 8 weeks of supervised exercise training.

While epigenetics refers to the study of single genes or sets of genes, epigenomics refers to global analyses of epigenetic changes across the entire genome. In this study, we utilized a high-resolution global DNA methylation analysis to explore the overall pattern of changes in DNA methylation across the genome following exercise intervention. We used the Illumina 850 K array to identify global patterns of changes in DNA methylation in metabolically well-characterized participants who underwent euglycemic hyperinsulinemic clamps combined with muscle biopsies before and after the exercise training program.

## 2. Results

### 2.1. Participants

Table 1 shows the characteristics of the sedentary study volunteers who took part in the study.

A total of 13 people took part in the study (5 males and 8 females). The average age across the participants was 34.6 ± 11.1, with a range of 21–54 years. On average, the participants had a body mass index (BMI) of 30.7 ± 7.4 kg/m^2^ and a body fat of 32.8 ± 7.6% prior to starting the training program. As shown in Table 1, the average fasting plasma glucose, fasting serum insulin, and hemoglobin A1c (HbA1c) were 95.5 ± 13.2 mg/dL, 8.3 ± 6.3 uIU/mL, and 5.38 ± 0.31 before entering into the training program.

### 2.2. Glucose and Insulin Metabolism

**Figure 1:**
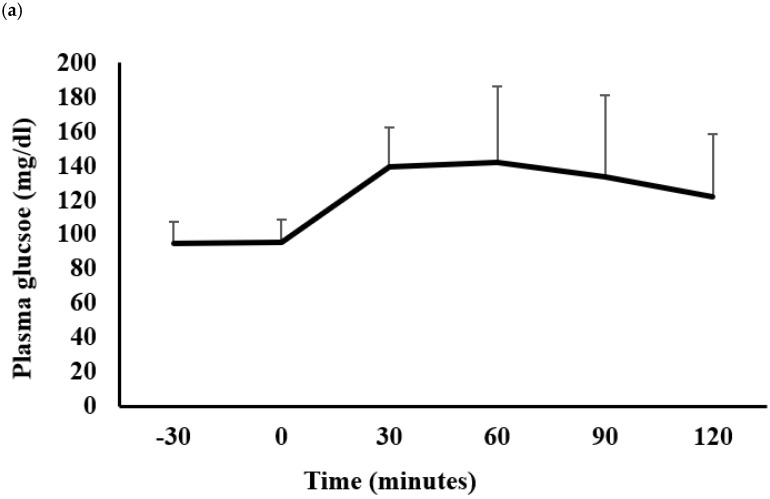

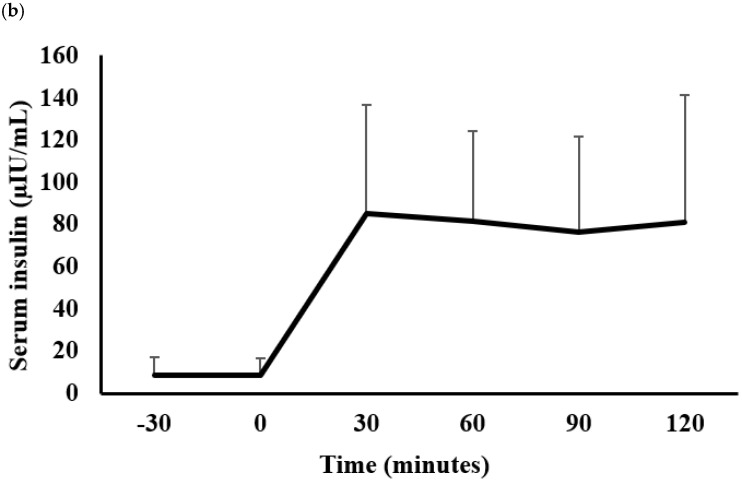
Glucose and insulin metabolism in the study volunteers. Plasma glucose (**a**) and serum insulin (**b**) responses to a 75 g oral glucose load. Data are mean ± SD.

Figure 1a,b show the plasma glucose and serum insulin responses to a 75 g oral glucose challenge, respectively. The oral glucose tolerance test findings show that the participants did not have diabetes. However, variability in the plasma glucose and serum insulin responses was observed, which was likely due to the participants’ large range of BMIs (20.8–46.9 kg/m^2^).

### 2.3. Training Improved Insulin Action

**Figure 2:**
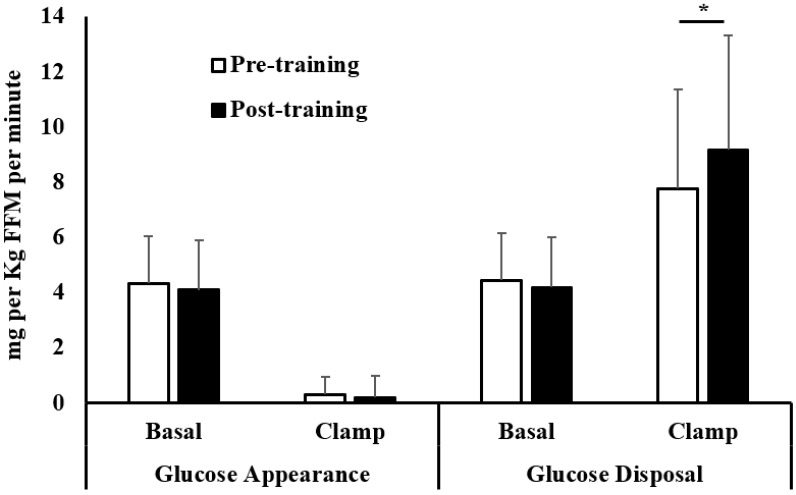
Basal and insulin-stimulated (clamp) rates of endogenous glucose appearance and glucose disposal determined using 6,6 di-deuterated glucose in the pre- versus post-training. Data are mean ± SD and expressed as mg per Kg FFM per minute. * *p* < 0.01, paired Student’s *t*-test.

We performed euglycemic hyperinsulinemic clamps to measure insulin action on glucose utilization in our participants before and after the 8-week exercise training. Figure 2 shows the rates of glucose appearance and disposal at basal and insulin-stimulated states, expressed in mg per Kg FFM per minute.

Rates of glucose appearance were similar pre- versus post-training basally (pre: 4.3 ± 1.7 versus post: 4.1 ± 1.8 mg per Kg FFM per minute, *p* = NS) and after insulin stimulation during the clamp (pre: 0.3 ± 0.6 versus post: 0.2 ± 0.8 mg per Kg FFM per minute, *p* = NS). We showed that rates of glucose disposal were similar in the pre- versus post-training participants in the basal state (pre: 4.4 ± 1.7 versus post: 4.2 ± 1.8 mg per Kg FFM per minute, *p* = NS). Following the exercise training, we observed a significant increase in insulin-stimulated glucose disposal (pre: 7.8 ± 3.6 versus post: 9.2 ± 4.1 mg per Kg FFM per minute, *p* < 0.01). It is important to note that there was a large range of insulin sensitivities in our cohort of participants, as measured by the glucose disposal insulin-stimulated (clamp); ranges pre-training: 1.98–13.45 and ranges post-training: 2.22–15.29 mg per Kg FFM per minute.

**Figure 3:**
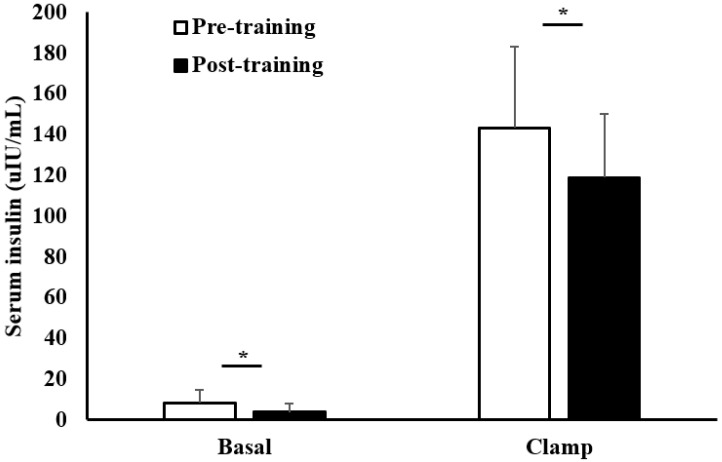
Fasting plasma insulin and serum insulin during the steady-state period of the clamp in the pre- versus post-training. Data are mean ± SD. * *p* < 0.001, paired Student’s *t*-test.

Figure 3 shows lower fasting serum insulin after the training (pre: 8.3 ± 6.3 versus post: 3.9 ± 4.4 uIU/mL, *p* < 0.001). In addition, serum insulin levels were significantly lower during the steady state of the clamp following the training (pre: 143.3 ± 40.0 versus post: 118.9 ± 31.1 uIU/mL, *p* < 0.001).

Moreover, insulin clearance rates during the clamp were increased following the training (pre: 0.63 ± 0.18 versus post: 0.77± 0.20 L per minute per m^2^, *p* < 0.05). Exercise training did not significantly change fasting plasma glucose levels, although there was a trend toward a reduction after training (pre: 95.5 ± 13.2 versus post: 90.6 ± 8.8 mg/dL, *p* = NS). Table 1 shows that training did not change the participant’s weight (pre: 87.5 ± 24.1 versus post: 87.0 ± 23.3 kg, *p* = NS). We expected stable weight, since we instructed participants to maintain their normal diet throughout the study.

### 2.4. Training Increased Peak Aerobic Activity

All participants adhered to the training program, as described in the Methods. We performed a peak aerobic activity (VO2 peak) test before and after the exercise training. Table 2 shows that exercise training significantly increased peak aerobic capacity from pre: 21.4 ± 4.1 to post: 24.7 ± 5.4 mL per Kg per minute, *p* < 0.0001. In addition, Table 2 shows that exercise training resulted in higher maximum heart rates and maximum workloads achieved during the VO2 peak test (*p* < 0.01 and *p* < 0.001, respectively).

### 2.5. Training Effects on Global DNA Methylation

After performing quality control (as described in the Methods), we performed differential methylation analysis on 844,280 probes. There was no difference in averaged global methylation (mean± standard deviation) in the pre- versus post-training (pre: 55.29 ± 30.09 versus post: 55.32 ± 29.8%, *p* = NS).

### 2.6. Training Effects on Individual DNA Methylation Sites

**Figure 4:**
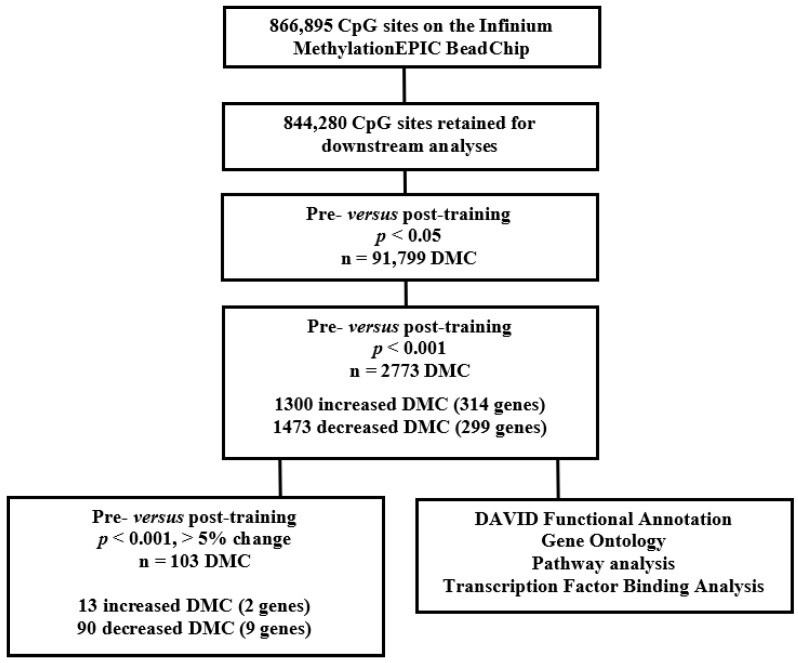
Workflow of methylation analysis. DMC = differentially methylated cytosine.

A flowchart of the methylation analysis is shown in Figure 4. No individual cytosine showed a significant difference in DNA methylation between the pre- versus post-training after a Benjamini–Hochberg multiple testing correction. Exercise training resulted in 91,799 differentially methylated cytosines (DMCs) using an uncorrected *p* < 0.05. Using an uncorrected *p* < 0.05 may result in false positive DMCs. As such, we filtered the data by an uncorrected *p* < 0.001. We selected a threshold of *p* < 0.001, in part, to identify DMCs that altered with training but also to reduce the false positives in the data, which may have occurred with a less stringent *p* < 0.05. Importantly, an unadjusted *p* value < 0.001 or less stringent at <0.05 have been used in other similar genome-wide methylation studies [8,10,11]. Using these criteria, 2773 DMCs significantly changed in response to 8 weeks of exercise (methylation mean change range of 0.5 to 9.3%). Of the 2773 DMCs, 1300 were increased (Table S1) and 1473 were decreased (Table S2) post-training.

To identify biologically meaningful sites, we filtered the 1300 increased and 1473 decreased DMCs on a mean change of >5% between the means of the pre- versus post-training. This resulted in 13 increased and 90 decreased DMCs post-training. Of the 13 increasers, 2 were assigned to the following genes: Follistatin-like 3 (*FSTL3*) and a long non-coding RNA (*RP11-624M8.1*) (Table 3).

Of the 90 decreasers, 9 were assigned to the following genes (Table 3): Cyclic Nucleotide Gated Channel Subunit Alpha 1 (*CNGA1*), Fc Fragment of IgG Receptor IIa (*FCGR2A*), Kinesin Family Member 21A (*KIF21A*), Meis Homeobox 1 (*MEIS1*), 5′-Nucleotidase Domain Containing 1 (*NT5DC1*), Olfactory Receptor Family 4 Subfamily D Member 1 (*OR4D1*), Pre-mRNA Processing Factor 4B (*PRPF4B*), Solute Carrier Family 26 Member 7 (*SLC26A7*), and Zinc Finger Protein 280C (*ZNF280C*). The data trended in the same direction for both females and males (Table S9) when we separated the 11 DMCs (that were assigned to genes) by sex.

### 2.7. Annotation Analysis of the Exercise Responsive DMC Genes

We performed the Database for Annotation, Visualization, and Integrated Discovery (DAVID) functional annotation analysis on the 1300 increased DMCs (Table S1) and 1473 decreased DMCs (Table S2). We used the DAVID analysis to determine whether the DMCs within genes enriched in pathways, gene ontology terms and identify potential transcription factor binding sites.

DAVID analysis of the increased DMC genes post-training revealed enrichment in 10 pathways, including h_mitrPathway: Signal Dependent Regulation of Myogenesis by Corepressor MITR, R-HSA-975110: TRAF6 mediated IRF7 activation in TLR7/8 or 9 signaling, R-HSA-2979096: NOTCH2 Activation and Transmission of Signal to the Nucleus and R-HSA-2173795: Downregulation of SMAD2/3: SMAD4 transcriptional activity (Table S3). Moreover, multiple gene ontology categories were enriched in the increasers, including GO:0007219~Notch signaling pathway, GO:0005667~transcription factor complex, GO:0007179~transforming growth factor beta receptor signaling pathway, GO:0045944~positive regulation of transcription from RNA polymerase II promoter, and GO:0071168~protein localization to chromatin (Table S4). In addition, many potential transcription factors (TFs) were enriched in the increased DMC genes, including the most significant TF *MYOD* (Table S5).

DAVID analysis of the decreased DMC genes post-training revealed enrichment in 14 pathways, including hsa04152: AMPK signaling pathway, hsa04071: Sphingolipid signaling pathway, h_rasPathway: Ras Signaling Pathway, h_ptdinsPathway: Phosphoinositides and their downstream targets (Table S6). Additionally, many gene ontology categories were enriched in the decreasers, including GO:0044822~poly(A) RNA binding, GO:0031491~nucleosome binding, GO:0035091~phosphatidylinositol binding, GO:0005871~kinesin complex, and GO:0051015~actin filament binding (Table S7). In addition, there were multiple potential transcription factors enriched in the decreased DMC genes, including NF-kappaB (NF-kB) (Table S8).

### 2.8. Training Effects on DNA Methyltransferase (DNMT) Gene Expression

DNA methyltransferases (DNMTs) tightly regulate methylation. Therefore, we sought to determine whether *DNMT1*, *DNMT3A,* and *DNMT3B* gene expression was altered following the training program. The quantitative RT-PCR results showed no change in mRNA expression for *DNMT1* pre- versus post-training (pre: 1.0 versus post: 1.3-fold change, *p* = NS). Likewise, there was no change in mRNA expression for *DNMT3A* (pre: 1.0 versus post: 0.99-fold change, *p* = NS) or *DNMT3B* (pre: 1.0 versus post: 1.3-fold change, *p* = NS).

### 2.9. Relationships between Exercise-Induced Methylation Change and Exercise-Induced Insulin Sensitivity Changes

Since we had a wide range of insulin sensitivities in our participants, we wanted to determine whether there were relationships between exercise-induced methylation change and exercise-induced insulin sensitivity change. Therefore, we performed correlation analyses between exercise-induced methylation changes in the 11 DMCs shown in Table 3 with the exercise-induced insulin-stimulated glucose disposal changes obtained from the hyperinsulinemic-euglycemic clamp. Linear regression analyses demonstrated no significant correlations (Table S10).

## 3. Discussion

The overarching goal of the present study was to determine whether epigenetic changes occur in skeletal muscle following exercise training. Specifically, this study measured DNA methylation in muscle following an 8-week aerobic exercise program in study volunteers with varying degrees of ages, body mass, and insulin sensitivities. In addition, this study described the metabolic changes observed following a supervised training regimen.

As expected, we demonstrated improvements in peak aerobic capacity following the exercise training. We [12,13,14] and others [15,16] previously showed that 8 weeks of aerobic exercise training significantly improves peak aerobic capacity. Additionally, the observed change in insulin-stimulated glucose disposal rate was consistent with previous exercise training protocols of 8 weeks [12,13,14]. The training effect on fasting serum insulin was striking; there was an over 60% decrease change in fasting insulin levels after training. In addition, we showed that insulin levels during the steady-state period in the clamp were much lower following the exercise training, reflecting the increased insulin clearance rates. We [17] and others [18] previously showed improvements in insulin levels and insulin clearance following an exercise intervention. Although these metabolic findings are not novel, we confirmed that the exercise training was successful in our cohort of participants.

As part of this study, we measured averaged global methylation across all cytosines following exercise training. We hypothesized that averaged global methylation pre- versus post-training would be unchanged following the training. We [19] and others [20] have performed methylation studies with and without various interventions and showed minimal to no changes at the global level. We showed a similar finding here in the present study. Although there were no changes at a global level, we identified novel DMCs that were changing with the exercise intervention using several filtering methods. Additionally, the DMCs identified appear to not be caused by changes in the mRNA expression of DNMTs, which were unaffected by the training. However, we did not measure DNMTs protein abundance, activity, or protein modifications, which may be affected by the training. Future studies are needed to address the effect of training on DNMTs.

Our approach was to filter DMCs with a methylation change of greater than 5% between the means of the groups. We performed this additional filter as others in the field used this approach [7,21] to identify DMCs that have biologically meaningful changes. Using these criteria, we identified 103 DMCs, with the majority decreased in methylation after training (87%), with the remainder increased. Interestingly, other exercise training studies showed similar trends, with more genes decreased in methylation following exercise training [6,8]. Collectively, this suggests less methylation in the promoters of genes, thereby increasing gene expression. Although we did not measure gene expression levels as part of this study, we previously showed that more genes increased in expression than decreased following an acute exercise protocol [22]. Another interesting finding from this study and others [2,6,7,8,9] showed that the mean methylation change was modest, with less than 10% following the training. However, whether the degree of methylation change is important to transcriptional events is unknown at this time.

To date, there have been several studies that have investigated DNA methylation in skeletal muscle following exercise training [6,7,8,9]. A 6-month training study in males with and without a family history of type 2 diabetes showed decreased DNA methylation of genes with known function in muscle and type 2 diabetes, including *MEF2A*, *RUNX1*, *NDUFC2*, and *THADA,* decreased after exercise [6]. In addition, 3 months of endurance training in healthy volunteers showed significant methylation changes in genes that code for muscle remodeling [9], inflammatory/immunological processes, and transcriptional regulation [9]. In another study, a 7-week training intervention in young males showed skeletal muscle possesses an epigenetic memory of earlier acute and chronic anabolic stimuli when encountering muscle hypertrophy [8]. Lastly, a 3-month training in young and old participants showed insignificant changes in gene promoter methylation [7]. There is no overlap between the DMCs identified in this study and the previous works described above. We speculate that this is due to differences in methodologies used, length and type of the exercise training program, and the differences in ages, BMIs, and sexes across the studies.

We identified several pathways from our methylation analyses that warrant a discussion. For the decreased DMCs, we observed gene enrichment in metabolic and actin-cytoskeleton pathways, including AMPK signaling, Ras signaling, phosphatidylinositol binding, actin filament binding, and kinesin complex. Previous work from our lab [12,14,17,22,23] and reviewed by others [1,24] showed that genes coding for proteins involved in metabolic pathways and the structure and function of the muscle itself might be involved in the metabolic improvements observed with exercise. We identified several interesting pathways for the increased DMCs, including signal-dependent regulation of myogenesis, *NOTCH2* activation and transmission, and *SMAD2/3: SMAD4* transcriptional activity. The *NOTCH* and *SMAD* signaling pathways are both required for satellite cell myogenesis and myogenic differentiation [25,26], so the idea that regulation of gene expression by changes in methylation in these pathways might regulate muscle remodeling during exercise training is intriguing. Additional work is necessary to understand the effect of DNA methylation on downstream transcriptional and translational events on these myogenic genes/pathways.

In addition to the pathways described above, we identified several candidate genes (*FSTL3*, *RP11-624M8.1*, *CNGA1*, *FCGR2A*, *KIF21A*, *MEIS1*, *NT5DC1*, *OR4D1*, *PRPF4B*, *SLC26A7*, and *ZNF280C*) that merit a discussion. Firstly, *FSTL3* is one of our most intriguing findings, in part, because of its emerging role in metabolic diseases [27]. *FSTL3* is a circulating glycoprotein expressed in various tissues, including skeletal muscle [28]. It is known to form complexes with TGF-β family members, including activin A and myostatin, to inhibit their function [29,30]. Circulating *FSTL3* levels are elevated in obese participants and associate with fat mass and markers of inflammation [31]. Additionally, a study of patients before and after bariatric surgery showed that circulating *FSTL3* was reduced following surgery [32]. Other studies suggest that *FSTL3* regulates body composition, glucose homeostasis, and islet function [33,34]. Moreover, *FSTL3* KO mice have increased pancreatic islet number and size, beta-cell hyperplasia, decreased visceral fat mass, improved glucose tolerance, and enhanced insulin insensitivity [33]. Clearly, this gene is important in metabolism, and future studies in insulin-resistant muscle with various interventions are needed. Other genes to highlight include *FCGR2A* that controls the immune response and plays a role in the clearance of immune complexes by macrophages, neutrophils, and platelets [35]. *KIF21A* is a subfamily member of the kinesin-like motor proteins and may be involved in microtubule-dependent transport [36]. *MEIS1* is involved in adipogenic differentiation processes [37]. Lastly, *ZNF280C* is a transcription factor that is a vital regulator for muscle tissue [38]. Additional studies are critical to determine whether these genes are important to the metabolic improvements observed following an exercise training program.

There are additional limitations that apply to this study. Firstly, a significant limitation of the present study is that we did not perform validation experiments on the genes identified from the methylation analysis. Secondly, there was the small number of participants studied, and we had insufficient numbers to analyze the data by sex. Thirdly, the study participants had a wide range of ages, BMI, and insulin sensitivity. Despite these limitations, we identified several pathways/genes enriched and differentially methylated following the training program. Moreover, we identified potential genes that may play a role in the metabolic improvements observed in skeletal muscle following exercise training. Additional studies for the future include studying a larger number of participants to tease apart the insulin sensitivity effects on methylation while analyzing by age, sex, and BMI. Another interest would be to utilize a training regimen with a longer time frame (6 months) and include a strength training component. Deciphering the role of detraining on methylation would be of interest too. Importantly, additional studies are warranted to address whether the degree of methylation change is important to transcriptional events in our genes of interest.

## 4. Materials and Methods

### 4.1. Participants

Thirteen sedentary participants were recruited in this study. None of the participants engaged in regular exercise. In addition, none of the participants reported a change in body weight for at least 3 months before entering the study. Participants were not taking any medication known to affect glucose metabolism. Participants were instructed not to exercise for 48 h prior to their screening, VO2 peak, and euglycemic hyperinsulinemic clamp visits, and to maintain their usual diet throughout the study. All subjects gave informed written consent to participate in the study, which was approved by the Institutional Review Boards of Arizona State University and University of Arizona.

### 4.2. Study Design

**Figure 5:**
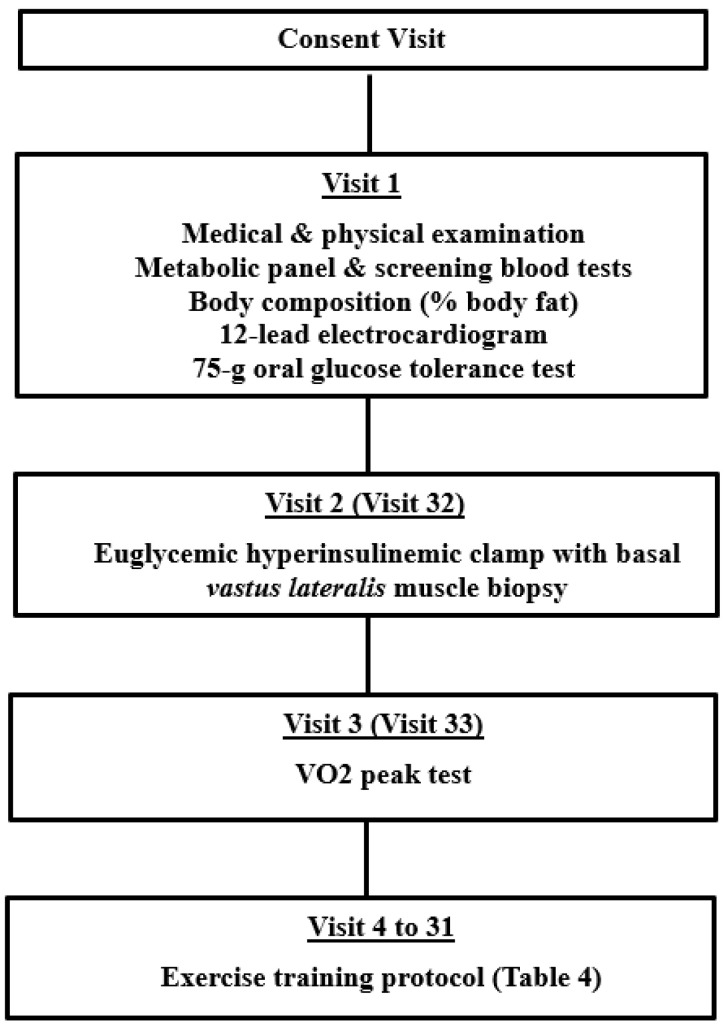
Outline of study design and visits.

Following a 10–12 h overnight fast, participants reported to either the Clinical Studies Infusion Unit (CSIU) at the Mayo Clinic Arizona or the Clinical and Translational Sciences Research Center (CATS) at the University of Arizona. A medical history, physical examination, 12-lead electrocardiogram, metabolic panel, and screening blood tests were obtained. Body composition was assessed by body impedance analysis (BioMarkers 2000). A 75 g oral glucose tolerance test was performed as per the American Diabetes Association criteria. After completing the screening studies, each participant had a euglycemic hyperinsulinemic clamp with muscle biopsy and, on a separate day, a peak aerobic activity test. Each participant then took part in an 8-week supervised exercise training program. At the end of the 8 weeks of training, a euglycemic hyperinsulinemic clamp with muscle biopsy and determination of VO2 peak were repeated. The study design is outlined in Figure 5.

### 4.3. Euglycemic Hyperinsulinemic Clamp

Participants reported fasting to either the CSIU or CATS to undergo an 80 mU/m^2^ euglycemic hyperinsulinemic clamp with muscle biopsy, as previously described [19,39]. Briefly, a primed infusion of 6,6 di-deuterated glucose was started at −120 min to determine the basal rate of glucose metabolism. Sixty minutes after the start of deuterated glucose infusion, a resting, basal *vastus lateralis* muscle biopsy was performed percutaneously, under local anesthesia. After resting for 1 h, a primed continuous infusion of insulin was started. The constant infusion of deuterated glucose was discontinued at 15 min after the start of the insulin infusion, and a variable infusion of 20% dextrose that was enriched with 6,6 di-deuterated glucose was used to maintain euglycemia and a constant enrichment of the tracer. The rates of glucose appearance and disappearance were calculated using steady-state equations to derive insulin sensitivity levels, termed the M value [39]. The insulin clearance rate during the clamp was calculated as the insulin infusion rate/(steady-state insulin during clamp–basal serum insulin).

### 4.4. Peak Aerobic Activity Test

Peak aerobic activity (VO2 peak) was determined using an electrically braked cycle ergometer and a Sensormedics model V29 Metabolic Measurement System (Sensormedics, Savi Park, CA, USA), as previously described [12]. Briefly, exercise was started at a workload of 40 W and increased by 10 W/min until perceived exhaustion or a respiratory quotient of 1.2 was reached. Heart rate and rhythm were monitored using a 12-lead electrocardiogram.

### 4.5. Exercise Training Program

All participants completed an 8-week aerobic exercise training program on a recumbent cycle (Schwinn 205P, Vancouver, WA, USA), as previously described [12]. All exercise sessions were supervised by one of the clinical team members. Participants initially exercised at 60% of their VO2 peak for 20 min on a stationary cycle ergometer 3 times per week. Over the course of the 8 weeks, exercise intensity, duration, and frequency were progressively increased to 70% of VO2 peak, for 45 min, 4 times per week (Table 4). Heart rate was used as an indicator of exercise intensity, with subjects exercising at a heart rate corresponding to the appropriate training VO2 peak.

### 4.6. Substrate and Hormone Determinations

Sonora Quest performed the screening laboratory tests and metabolic panel. Plasma glucose concentration was determined by the glucose oxidase method on the GM9 Glucose Analyser (Analox Instruments, Stourbridge, UK). Serum insulin was measured by an ELISA (Alpco, Salem, NH, USA). The enrichment of plasma glucose with 6,6 di-deuterated glucose was assayed using GC/MS in the Center for Clinical and Translational Science (CCaTS) Metabolomics Core at the Mayo Clinic in Rochester.

### 4.7. Muscle Biopsy Processing

Homogenization of the *vastus lateralis* muscle biopsy (25 mg) was performed in 1X PBS using a Polytron (Brinkmann Instruments Westbury, New York, NY, USA). DNA was isolated using QIAamp DNA mini kit, as per the manufacturer’s instructions (Qiagen, Valencia, CA, USA). DNA quality and quantity were assessed using agarose gel electrophoresis and spectrophotometer A260/A280 values, as determined using the NanoVue (GE Healthcare, Buckinghamshire, UK).

### 4.8. Global DNA Methylation and Analysis

DNA samples were bisulfite converted using the EZ DNA Methylation Kit (Zymo Research, Irvine, CA, USA), as per the manufacturer’s protocol. Global DNA methylation was measured using the Illumina Infinium MethylationEPIC BeadChip (Illumina, San Diego, CA, USA), as per the manufacturer’s protocol. The Infinium MethylationEPIC BeadChip interrogates over 850,000 methylation sites across the human genome, covering 99% of RefSeq genes. The 850K EPIC array data were preprocessed using RnBeads package (version 2.2.0) under R version 3.6.0. A total of 17,371 sites were removed, since they overlapped with single nucleotide polymorphisms (SNPs). An additional 2262 probes were removed because of unreliable measurements, as determined by the Greedycut algorithm as implemented in RnBeads. Data normalization was performed using the algorithm implemented in the wateRmelon package. A second filter step was carried out after data normalization, which retained 844,280 probes for downstream analysis. Differential methylation analyses were performed using the generalized linear model from limma package and adjusted for predicted sex. A paired measurement design was implemented for the pre- versus post-training methylation levels. A Benjamini–Hochberg multiple testing correction yielded no significant DMCs, therefore, an uncorrected *p* < 0.001 was used. Additionally, the DMCs were filtered on a methylation change of >5% between the means of the group. Figure 4 illustrates a summary of the methylation analysis. The methylation dataset supporting the conclusions of this article is available in ArrayExpress (https://www.ebi.ac.uk/arrayexpress/help/index.html (accessed on 10 December 2021)) accession number E-MTAB-11282.

### 4.9. Pathway Analysis and Predicted Transcription Factor Binding Analysis

The Database for Annotation, Visualization, and Integrated Discovery (DAVID) pathway analysis was performed on the differentially methylated cytosines (defined as uncorrected *p* < 0.001). Transcription factor binding sites analysis was performed on the significant DMCs using DAVID analysis.

### 4.10. DNA Methyltransferases (DNMT) Quantitative RT-PCR

Skeletal muscle gene expression for *DNMT1*, *DNMT3A,* and *DNMT3B* was detected using quantitative Real-Time PCR on the ABI PRISM 7900HT sequence detection system (Life Technologies, Carlsbad, CA, USA). TaqMan Universal Fast PCR master mix reagents and the Assay-On-Demand gene expression primer pair and probes (Life Technologies, Carlsbad, CA) were added to 100 ng cDNA, which was synthesized using the ABI High Capacity cDNA Reverse Transcription Kit, as per manufacturer’s instructions. The quantity of *DNMT1* (Hs00945890_m1), *DNMT3A* (Hs00601097_m1), and *DNMT3B* (Hs01003405_m1) in each sample was normalized to *18S* (Hs99999901_s1) using the comparative (2^−ΔΔCT^) method [40].

### 4.11. Statistical Analysis

Data were expressed as mean ± standard deviation (SD). Statistical comparisons of the pre- versus post-training characteristic data were performed using a paired Student’s *t*-test. The significance level was set at *p* < 0.05. Linear regression analyses were performed using STATA 14 (StataCorp, College Station, TX, USA). The analysis of global DNA methylation is described above.

## 5. Conclusions

**Figure 6:**
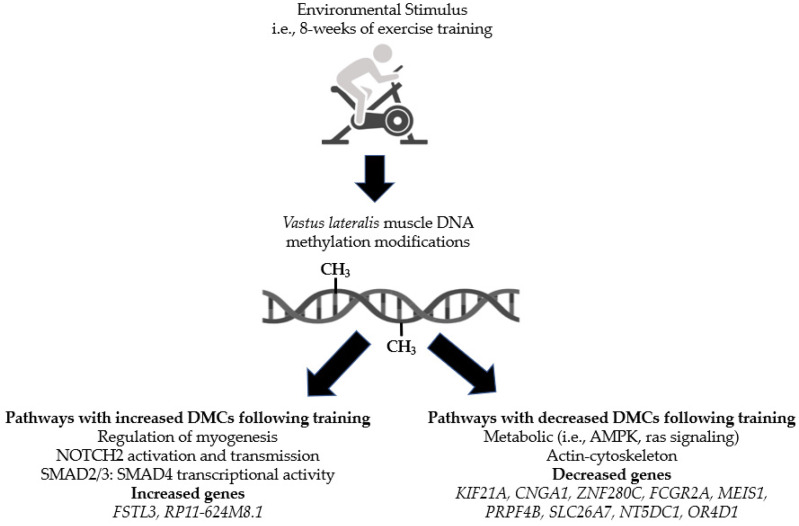
Summary of findings.

Our findings showed that 8 weeks of exercise training alters skeletal muscle DNA methylation of specific genes and pathways in people with varying degrees of insulin sensitivity. Although, we did not observe changes at the global level, we did identify a number of genes and pathways for future investigations. Our study also provides additional evidence that an environmental stimulus, such as physical activity, can alter epigenetic processes, specifically DNA methylation. The conclusions of our study are summarized in Figure 6.

## Figures and Tables

**Table 1:** Characteristics of volunteers who participated in the study.

	Pre-Training	Post-Training	*p* Value
Male/Female	5 M/8 F		
Age (years)	34.6 ± 11.1 (21–54)		
Body mass index (kg/m^2^)	30.7 ± 7.4 (20.8–46.9)	30.6 ± 2.0 (21.4–46.2)	NS
Body fat (%)	32.8 ± 7.6 (16.6–45.8)	32.9 ± 7.4 (17.8–46.2)	NS
Weight (kg)	87.5 ± 24.1 (48.8–134.8)	87.0 ± 23.3 (50.1–132.7)	NS
Fat mass (kg)	29.5 ± 13.1 (12.6–61.8)	29.3 ± 12.9 (12.9–61.3)	NS
Fat free mass (kg)	58.0 ± 14.1 (36.2–81.6)	57.7 ± 13.7 (36.8–79.4)	NS
Hemoglobin A1c %	5.38 ± 0.31 (4.70–5.80)	Nd	-
Fasting plasma glucose (mg/dL)	95.5 ± 13.2 (79.4–133.5)	90.6 ± 8.8 (74.3–103.0)	NS
Fasting serum insulin (uIU/mL)	8.3 ± 6.3 (0.6–24.5)	3.9 ± 4.4 (0.1–17.4)	<0.001
Total cholesterol (mg/dL)	168.4 ± 28.1 (126.0–208.0)	Nd	-
Plasma triglycerides (mg/dL)	112.2 ± 66.9 (31.0–265.0)	Nd	-
Low-density lipoprotein (mg/dL)	93.0 ± 22.7 (53.0–130.0)	Nd	-
High-density lipoprotein (mg/dL)	53.0 ± 15.9 (30.0–87.0)	Nd	-
Systolic blood pressure (mmHg)	122.5 ± 13.6 (97.0–145.0)	121.1 ± 11.2 (101.0–143.0)	NS
Diastolic blood pressure (mmHg)	73.3 ± 8.4 (61.0–90.0)	72.5 ± 10.3 (61.0–90.0)	NS

Data are presented as mean ± SD. Minimum–Maximum values shown in parentheses. Nd = no data; not measured post-training.

**Table 2:** Effect of aerobic exercise training on exercise capacity.

	Pre-Training	Post-Training	*p* Value
Resting heart rate (beats/minute)	82.9 ± 9.8	80.1 ± 7.6	NS
Maximum heart rate (beats/minute)	163.8 ± 15.3	171.9 ± 15.9	<0.01
Maximum RER	1.24 ± 0.06	1.24 ± 0.06	NS
Maximum workload (watts)	155.6 ± 35.2	181.7 ± 34.8	<0.001
VO2 peak (ml per kg per minute)	21.4 ± 4.1	24.7 ± 5.4	<0.0001

Data presented as mean ± SD. Peak measurements are maximum values obtained during the peak aerobic activity (VO2 peak) test. Maximum heart rate is the heart rate achieved at peak aerobic activity. Maximum workload represents the highest workload reached and sustained, corresponding to the maximum HR and VO2 peak observed during VO2 peak test. RER = respiratory exchange ratio. The *p* values derived from the paired Student’s *t*-test.

**Table 3:** Differentially methylated cytosines (DMCs, *p* < 0.001 and >5% change) following exercise training.

Array ID	Methylation % Pre-Training	Methylation % Post-Training	Mean Difference	*p* Value	Gene Symbol
cg17850273	78.0	68.7	−9.35	<0.001	*KIF21A*
cg03825843	80.2	73.3	−6.85	<0.001	*CNGA1*
cg25445870	72.5	65.7	−6.77	<0.001	*ZNF280C*
cg27565811	71.2	64.9	−6.34	<0.001	*FCGR2A*
cg12999414	85.0	78.8	−6.16	<0.001	*MEIS1*
cg13101948	72.7	67.4	−5.37	<0.001	*PRPF4B*
cg06442162	81.8	76.4	−5.37	<0.001	*SLC26A7*
cg11637017	83.8	78.8	−5.01	<0.001	*NT5DC1*
cg12463722	64.1	59.1	−5.00	<0.001	*OR4D1*
cg22305455	38.3	43.3	5.01	<0.001	*FSTL3*
cg02797038	20.0	25.6	5.60	<0.001	*RP11-624M8.1*

Data presented as methylation percentages and organized by mean difference. Differential methylation analyses were performed using the generalized linear model from limma package and adjusted for predicted sex. A paired measurement design was implemented for the pre- versus post-training methylation levels. The *p* value is uncorrected.

**Table 4:** Exercise training program.

Week	% VO2 Peak	Time	Frequency
1	60%	20 min	3/week
2	60%	25 min	3/week
3	60–65%	30 min	3/week
4	60–65%	35 min	3/week
5	65%	35 min	4/week
6	65–70%	40 min	4/week
7	70%	45 min	4/week
8	70%	45 min	4/week

## Data Availability

The methylation dataset supporting the conclusions of this article is available in ArrayExpress (https://www.ebi.ac.uk/arrayexpress/help/index.html (accessed on 10 December 2021)) accession number E-MTAB-11282.

## Supplementary Materials

The following supporting information can be downloaded at: https://www.mdpi.com/article/10.3390/metabo12030222/s1, Table S1: Differentially methylated cytosines (*n* = 1330) significantly (*p* < 0.001) increased post-training; Table S2: Differentially methylated cytosines (*n* = 1473) significantly (*p* < 0.001) decreased post-training; Table S3: DAVID pathway analysis on the genes with significantly increased DMCs post-training; Table S4: DAVID gene ontology analysis on the genes with significantly increased DMCs post-training; Table S5: DAVID transcription factor binding site (TFBS) analysis on the genes with significantly increased DMCs post-training; Table S6: DAVID pathway analysis on the genes with significantly decreased DMCs post-training; Table S7: DAVID gene ontology analysis on the genes with significantly decreased DMCs post-training; Table S8: DAVID transcription factor binding site (TFBS) analysis on the genes with significantly decreased DMCs post-training; Table S9: Differentially methylated cytosines (DMCs, *p* < 0.001 and >5% change) following exercise training separated by females and males; Table S10: Linear regression analysis of % exercise-induced methylation changes with % exercise-induced insulin-stimulated glucose disposal changes.
